# The Buffalo Medical Journal

**Published:** 1845-07

**Authors:** 


The Buffalo Medical Journal." This is published at Buffalo, in the State of New York, and is edited by Austin Flint, M. D., a name not unknown in the journals of the country. The first number bears date on the first of June, 1845, is in octavo form, and contains twenty pages of original and selected matter. The specimen before us is creditably got up, in matter and manner, and promises well for the interest and instruction of its patrons. To one and all of these, our new cotemporaries, we cheerfully extend the right hand of fellowship, and in the honorable and useful task of disseminating medical knowledge, bid them God speed!

